# Systems Biology Modeling Reveals a Possible Mechanism of the Tumor Cell Death upon Oncogene Inactivation in EGFR Addicted Cancers

**DOI:** 10.1371/journal.pone.0028930

**Published:** 2011-12-14

**Authors:** Jian-Ping Zhou, Xin Chen, Shan Feng, Shi-Dong Luo, You-Li Pan, Lei Zhong, Pan Ji, Ze-Rong Wang, Shuang Ma, Lin-Li Li, Yu-Quan Wei, Sheng-Yong Yang

**Affiliations:** 1 State Key Laboratory of Biotherapy and Cancer Center, West China Hospital, Sichuan University, Chengdu, Sichuan, People's Republic of China; 2 Medical School, Panzhihua University, Panzhihua, Sichuan, People's Republic of China; Sun Yat-sen University Medical School, China

## Abstract

Despite many evidences supporting the concept of “oncogene addiction” and many hypotheses rationalizing it, there is still a lack of detailed understanding to the precise molecular mechanism underlying oncogene addiction. In this account, we developed a mathematic model of epidermal growth factor receptor (EGFR) associated signaling network, which involves EGFR-driving proliferation/pro-survival signaling pathways Ras/extracellular-signal-regulated kinase (ERK) and phosphoinositol-3 kinase (PI3K)/AKT, and pro-apoptotic signaling pathway apoptosis signal-regulating kinase 1 (ASK1)/p38. In the setting of sustained EGFR activation, the simulation results show a persistent high level of proliferation/pro-survival effectors phospho-ERK and phospho-AKT, and a basal level of pro-apoptotic effector phospho-p38. The potential of p38 activation (apoptotic potential) due to the elevated level of reactive oxygen species (ROS) is largely suppressed by the negative crosstalk between PI3K/AKT and ASK1/p38 pathways. Upon acute EGFR inactivation, the survival signals decay rapidly, followed by a fast increase of the apoptotic signal due to the release of apoptotic potential. Overall, our systems biology modeling together with experimental validations reveals that inhibition of survival signals and concomitant release of apoptotic potential jointly contribute to the tumor cell death following the inhibition of addicted oncogene in EGFR addicted cancers.

## Introduction

The concept of “oncogene addiction” was firstly raised by Weinstein based on the peculiar phenomena that the proliferation and survival of some cancers strongly depend on only one oncogenic protein or pathway, despite the presence of multiple gene mutations and epigenetic abnormalities [1]–[3]. Now a lot of evidences have been found to support this concept, including those from genetically engineered mouse models [4], [5], mechanistic studies in human cancer cell lines [6], [7], and particularly the good clinical therapeutic efficacy of a series of antibodies or small molecular drugs that target specific proteins in human cancers reported in recent years [8]–[10]. Currently several hypotheses have been proposed to explain the phenomenon of oncogene addiction, including genetic streamlining [11], [12], synthetic lethality [13], oncogenic amnesia [14], and oncogenic shock [15], [16]. These hypotheses give various explanations from different angles to the phenomenon of oncogene addiction. Even so, there is still a lack of detailed understanding to the precise mechanism underlying the oncogene addiction. In particular, the molecular basis of some essential phenomena related to the oncogene addiction remains unclear, for example, the phenomenon that acute oncogene inactivation leads to tumor cell death in the oncogene addicted cancers, while sparing other cells that are not similarly addicted.

It has been suggested that the abnormity of intracellular circuitry (signal transduction network) or “wiring diagram” is the most fundamental reason that accounts for the phenomena of oncogene addiction [2], [17]. The complexity of intracellular circuitry together with multi-genetic mutations in cancer cells hampers the understanding of molecular basis underpinning oncogene addiction [18], [19]. The situation has now changed a little due to recent advances in systems biology [20]–[22], particularly the computational systems biology [23], [24]. Thus, one is currently in a good position to apply such technologies to reveal possible molecular mechanisms underlying various phenomena associated with the oncogene addiction.

As the first work of understanding the oncogene addiction from the viewpoint of systems biology, in this study, we developed a mathematic model of epidermal growth factor receptor (EGFR)-associated signaling network to investigate possible molecular mechanism of the tumor cell death following the inhibition of addicted oncogene. Here we chose the EGFR-associated signaling network mainly due to the following reasons: (1) EGFR is one of the most important oncogenes and implicated in many human tumor types, in particular, lung cancers, head and neck tumors [25], [26]; (2) the EGFR signaling network has been widely studied experimentally and theoretically [27]–[30], implying that many parameters are available in literature that facilitates the model development. This model was validated first, and then utilized to simulate the normal state of cancer cells and network responses upon acute EGFR inhibition.

## Results

### Establishment of the mathematic model of EGFR-associated signaling network

**Figure 1:**
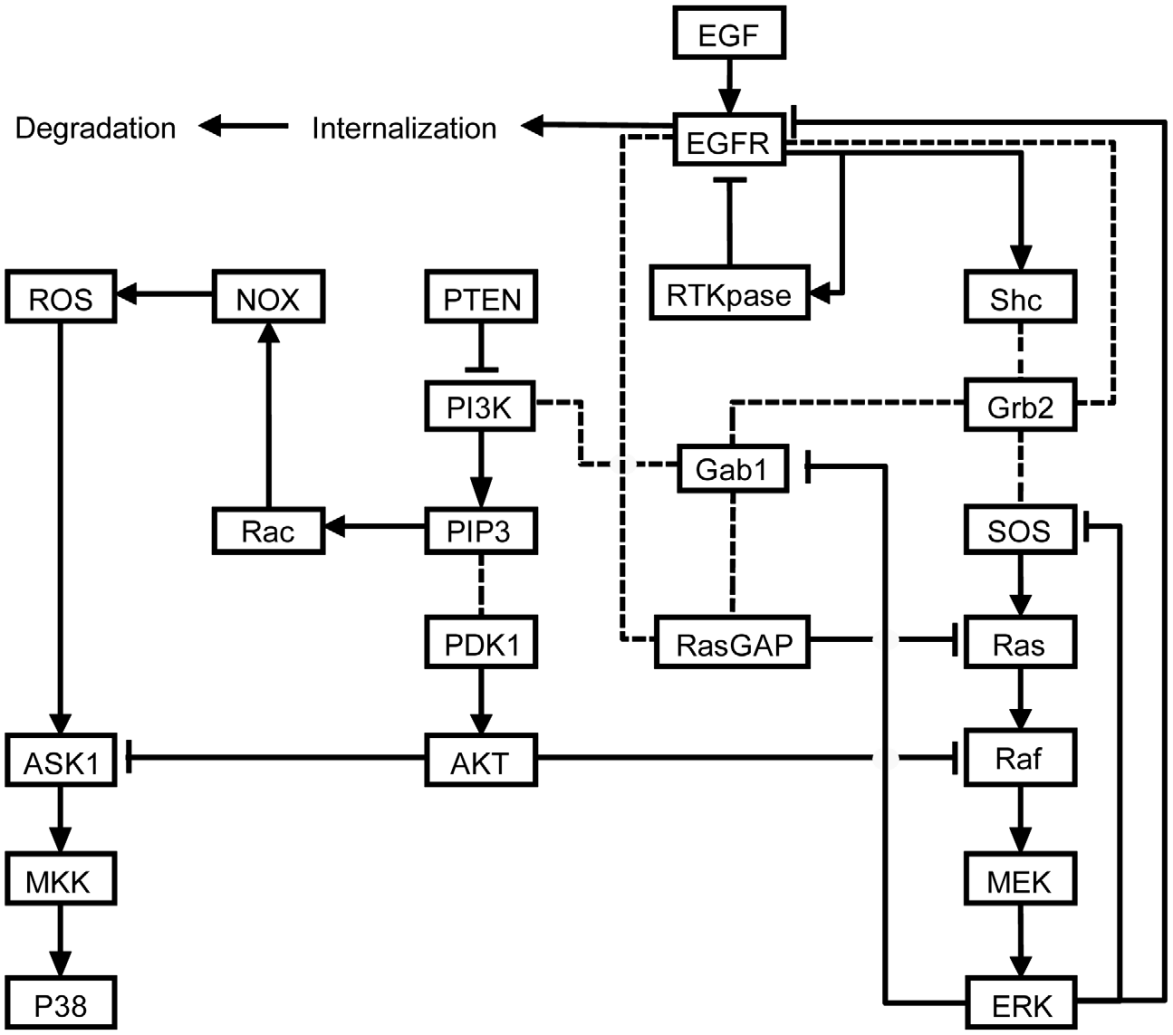
Simplified schematic representation of the EGFR-associated signaling network model established in this study. Solid lines with arrows indicate the activation of proteins or lipids. Dotted lines represent direct protein–protein and protein–lipid interactions. Solid lines with blunt ends represent inhibition.

We here present an ordinary differential equation (ODE) based mathematic model of EGFR-associated signaling network, which involves EGFR-driving proliferation/pro-survival signaling pathways Ras/extracellular-signal-regulated kinase (ERK) and phosphoinositol-3 kinase (PI3K)/AKT, and pro-apoptotic signaling pathway apoptosis signal-regulating kinase 1 (ASK1)/p38. The portions of Ras/ERK and PI3K/AKT pathways in this model were established based on the known Ras/ERK and PI3K/AKT models including such as Brightman [31], Birtwistle [30], Schoeberl [29], and Oda [32] models. To the authors' knowledge, however, there is no mathematic model of p38 mediated pro-apoptotic signaling pathway reported yet in literature. We thus built a model of p38 signaling and incorporated it into the EGFR signaling network. The model comprises 243 equations and interactions with 160 distinct molecular species, characterized by 145 kinetic parameters and 28 non-zero initial molecular concentrations. Most of the kinetic parameters and initial molecular concentrations in this model were taken from literature or derived from basic physicochemical quantities [29], [30], [33]. Others were estimated by fitting model outputs to known experimental data with the use of hybrid quasi ensemble modeling algorithm proposed by us recently [34]. The main reactions and parameters are presented in Table S1, and initial molecular concentrations in Table S2 (see Supporting Information). The important signaling pathways and key components involved in our network model are shown in Figure 1. A brief description for this EGFR-associated network is given as follows.

In the normal condition, the network signaling is initiated by binding of epidermal growth factor (EGF) to EGFR, followed by the dimerization and subsequent mutual trans-phosphorylation on several tyrosine residues of EGFR; although EGFR family has other three members including ErbB2, ErbB3, and ErbB4 in addition to EGFR (ErbB1) which can form different dimmers (either homo- or hetero-dimers) [30], [35], just EGFR and EGFR-EGFR homo-dimers are considered in this model for simplification. These phospho-tyrosine residues act as docking sites that enable receptors to recruit adaptor proteins Shc and Grb2 [36], which can then recruit the guanosine nucleotide exchange factor SOS. SOS promotes the replacement of GDP by GTP in Ras, thereby activating Ras [37]. The activated Ras subsequently results in the activation of protein kinase Raf [38]; since the direct activator of Raf kinase is not known yet, we assume that Raf phosphorylation is caused directly by a Ras-GTP molecule in this model. The activated Raf finally activates the mitogen-activated protein kinase kinase1/2 (MEK) and ERK in a cascade way [39]. The activated ERK can phosphorylate the upstream protein SOS, hence causing the dissociation of Grb2-SOS from the receptor complex, which forms a negative feedback loop [40]. In addition, Grb2 in cytomembrane can also recruit Gab1, which causes the phosphorylation of Gab1 and the consequent recruitment of PI3K [41]. In cytomembrane, PI3K transforms phosphatidylinositol 4,5-bisphosphate (PIP2) into phosphatidylinositol (3,4,5)-trisphosphate (PIP3) that induces the activation of AKT through cooperating with 3-phosphoinositide dependent protein kinase-1 (PDK1) [42]. In this process, phosphatase and tensin homolog (PTEN) and protein phosphatase 2A (PP2A) can specifically dephosphorylate PIP3 and AKT respectively [43]. Phosphorylated Raf, MEK and ERK can be dephosphorylated by their specific phosphatases [30].

P38 that is thought as an important pro-apoptotic effector can be activated by a variety of environmental stresses and inflammatory cytokines [44]–[46]. Among which, of particular importance is the stress due to reactive oxygen species (ROS). For example, Dolado et al. reported that the oncogenic H-Ras-induced ROS plays a key role in the inhibition of tumor initiation by activating p38 and hence resulting in apoptosis in fibroblasts derived from mouse embryos [47]. Moreover numerous studies have demonstrated that stimulation of human cancer cells with EGF results in an increase in the intracellular concentration of ROS [48]–[50]. Jin and his colleagues even detected the rate of ROS generation induced by EGF stimulation in A431 [50]. Thus, ROS is involved in our model as an important upstream component of p38 signaling pathway. ROS triggers the activation of ASK1, which then induces the phosphorylation of mitogen-activated protein kinase kinase3/6 (MKK) and p38 in a cascade style [51]. Specific phosphatases of ASK1, MKK and p38 are also included in this model. In addition, it has been established that activated AKT can suppress the activation of p38 via phosphorylating ASK1, which forms a negative crosstalk between PI3K/AKT and ASK1/p38 signaling pathways. For instance, Zhang et al. reported that AKT can phosphorylate ASK1 at site Ser83 to inhibit hydrogen peroxide-induced ASK1/p38 signaling activation in endothelial cells [52]. Yuan and his collegues also proved that AKT can inhibit the cisplatin-induced p38 activation through phosphorylation of ASK1 [53]. Accordingly, the crosstalk AKT-ASK1 between PI3K/AKT and ASK1/p38 signaling pathways is also involved in our model.

### Model validation

**Figure 2:**
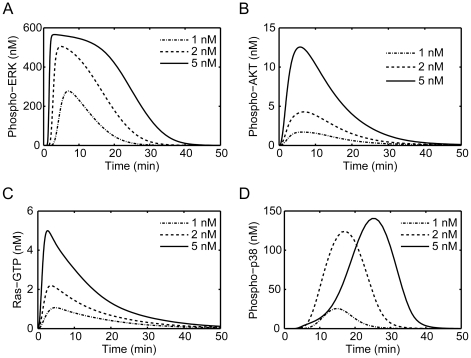
Simulation of the phosphorylation profiles of some critical signals under transient EGF stimulation. Concentration changes of (A) active ERK (phospho-ERK), (B) active AKT (phospho-AKT), (C) active Ras (Ras-GTP) and (D) active p38 (phospho-p38) upon transient stimulation by EGF at different concentrations.

The established model was validated by calculating the time courses of several key species activation including Ras, ERK, AKT and p38 after a short EGF stimulation and comparing the simulation results with other published experimental or simulation studies on EGFR signaling. Figure 2 shows the simulated time course patterns of Ras, ERK, AKT and p38 activation exposure to EGF with varying concentrations over 60 minutes. Upon addition of EGF at t = 0, the total concentrations of Ras-GTP, phospho-ERK (p-ERK) and phospho-AKT (p-AKT) rapidly increase to a maximum within 5 minutes with their peaks depending on the EGF concentration, followed by decaying to their basal levels within 50 minutes. Meantime, a delayed increase of phospho-p38 (p-p38), which is peaked after 15 minutes, is observed. These are consistent with previous experimental findings. For instance, EGF stimulation of PC12 cells results in a rapid, transient activation of Ras and ERK [27]. The level of Ras-GTP rapidly reaches a maximum within 2 minutes exposure to EGF, but declines within 10 minutes and gradually returns to the basal level within 60 minutes. Similarly, ERK activation is maximal within 5 minutes, and decays to less than 50% of the maximum level by 30 minutes, and to less than 25% within 60 minutes. The transient activations of ERK and p38 after EGF stimulation was also observed in retinal capillary endothelial cells [54]. After a 5-minute exposure of retinal capillary endothelial cells to EGF, ERK reaches a maximum level of phosphorylation that is 20-fold greater than that of its control. During the subsequent 2 hours, ERK activation decays until it reaches a baseline. For p-p38, it increases a level nearly threefold relative to its control after 15 minutes, followed by a decline to its control level.

### Simulation of sustained activation of EGFR

**Figure 3:**
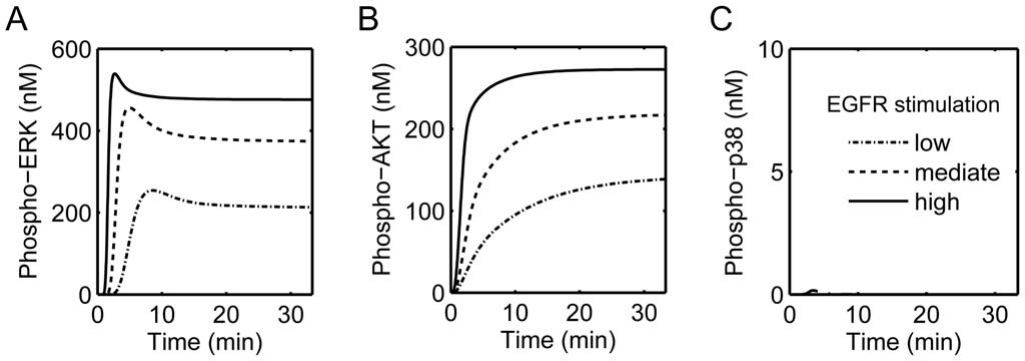
Simulation of the protein phosphorylation profiles at sustained activation of EGFR. Concentration changes of (A) active ERK (phospho-ERK), (B) active AKT (phospho-AKT) and (C) active p38 (phospho-p38) upon sustained stimulation of EGFR at different levels.

We then simulate the sustained EGFR activation in EGFR addicted cancer cells. For this purpose, the receptor internalization reactions were removed from the model, and the EGF concentration was set to a fixed value. Figure 3 presents the time courses of ERK, AKT and p38 activation. Clearly, the sustained EGFR activation finally results in a stable high level of p-ERK and p-AKT after a rapid increase in the beginning. However, p38 activation still keeps at its basal level although a detectable small peak of p-p38 within the first 5 minutes. Overall, the sustained activation of EGFR leads to a final result of persistent high level of p-ERK and p-AKT, but a low level of p-p38. This is consistent with experimental findings in EGFR addicted cancer cells that the ERK and AKT keep in a high level of activation and the p38 maintains in a low level of activation [16].

### Simulation of the network response upon acute EGFR inactivation in EGFR addicted cancer cells

**Figure 4:**
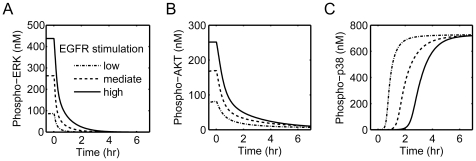
Simulation of the molecular responses upon acute EGFR inhibition. Profile of concentration changes of (A) active ERK (phospho-ERK), (B) active AKT (phospho-AKT) and (C) active p38 (phospho-p38). The virtual event of EGFR inhibition was performed at time 0 hr.

We have just simulated the normal state of EGFR addicted cancer cells, namely a setting of sustained EGFR activation. In this section, we shall simulate the network response upon acute EGFR inactivation, which mimics the situation of using the EGFR inhibitors in EGFR addicted cancer cells. In this simulation, the acute EGFR inactivation was carried out through a pre-assigned event at a fixed time point, namely the EGFR activity was set to its basal level. Changes in concentration of activated ERK, AKT and p38 after acute EGFR inhibition are shown in Figure 4. Obviously, the acute EGFR inactivation leads to an immediate decline of the concentration of p-ERK and p-AKT, and a delayed increase of p-p38. The simulation well reproduced the experimental phenomena that acute inactivation of EGFR oncogene results in a rapid diminution of the cell proliferation/pro-survival effectors p-ERK and p-AKT, and subsequent engagement of the pro-apoptotic effector p-p38 in EGFR addicted cancer cells [16], [55].

### Sensitivity analysis reveals critical factors responsible for the p38 activation

**Figure 5:**
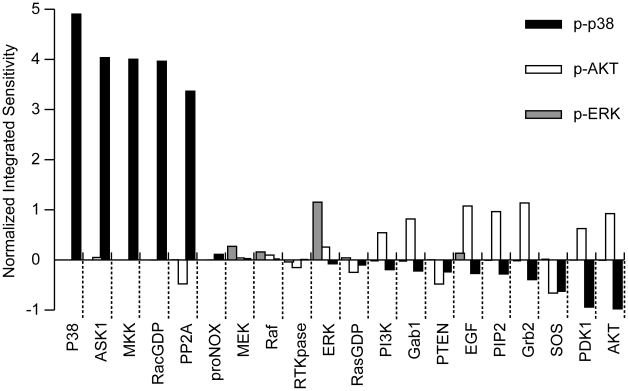
Sensitivity analysis of model species. Normalized time-integrated sensitivities of ERK, AKT and p38 phosphorylation (p-ERK, p-AKT, and p-p38) to each nonzero species in the EGFR-associated network model.

Sensitivity analysis has long been used in systems biology to study how variation of the value of the simulated output of a mathematical model can be apportioned to different sources of variation in the input of a model, which can be used to identify critical proteins that dominate the output of an effector protein [29]. Here p-ERK, p-AKT and p-p38 were taken as the output variables for the sensitivity analysis. Figure 5 presents the normalized time-integrated sensitivity for species with nonzero values in the EGFR signaling network model. Obviously, the most sensitive nodes for ERK activation are MEK and Raf in addition to the incurious ERK and EGF; ERK and EGF are the trivial solutions since ERK is the direct precursor of p-ERK, and EGF is the initiator of the network response (for simplicity, these incurious sensitive nodes will be ignored later). For AKT activation, the most sensitive nodes include Grb2, PIP2, Gab1, PI3K, PDK1, SOS, and PTEN. And those for the activation of p38 mainly include ASK1, MKK, RacGDP, PP2A, AKT, PDK1 and SOS.

**Figure 6:**
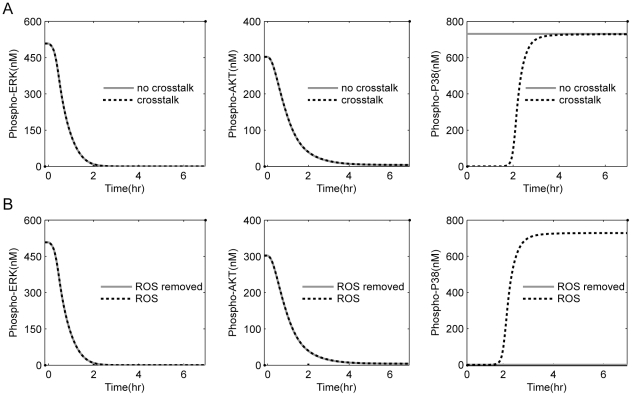
Influences of removing either the crosstalk AKT-ASK1 or ROS on the p-ERK, p-AKT and p-p38 levels. The virtual event of EGFR inhibition was performed at time 0 hr. (A) The effects of removing crosstalk AKT-ASK1 on the concentration changes of p-ERK, p-AKT and p-P38. (B) The effects of ROS removal on the concentration changes of p-ERK, p-AKT and p-P38.

The sensitivity analysis above reveals the critical regulators in the network for the survival effectors p-ERK and p-AKT, and pro-apoptotic effector p-p38. We also observed that the largest positive and negative values of sensitivity all correspond to the output variable p-p38. For example, AKT that has the largest negative value of sensitivity to the pro-apoptotic effector p-p38 may play an important negative regulation role in cell apoptosis. ASK1, MKK and RacGDP, which all are the key components of ROS/ASK1/MKK/p38 signaling cascade, have the largest positive values of sensitivity, implying a key positive role of ROS associated signaling in cell apoptosis. These findings may also be related to the sensitivity of tumor cells to the killing effects of targeted drugs. In order to test this hypothesis, we changed the original network by removing either the crosstalk AKT-ASK1 or ROS, and then re-simulated the network responses in the settings of sustained EGFR activation and acute EGFR inactivation. Figure 6A presents the simulation results when removing the crosstalk AKT-ASK1. Obviously, high levels of p-ERK and p-AKT in the setting of sustained EGFR activation and a rapid drop upon acute EGFR inhibition (see Figure 6A) were observed, which are very similar to the modeling results obtained on the original network. However, after removing the crosstalk AKT-ASK1, p-p38 keeps in a high level in the case of either sustained EGFR activation or acute EGFR inhibition, implying a stronger apoptotic capacity. Figure 6B depicts the modeling results when removing ROS. Similar to the case when removing AKT-ASK1, we still monitored high levels of p-ERK and p-AKT in the setting of sustained EGFR activation and a rapid drop upon acute EGFR inhibition. However, ROS removal resulted in a low level of p-p38 in the case of either sustained EGFR activation or acute EGFR inactivation. These data show that removal of ROS leads to the loss of apoptotic capacity.

Collectively, the simulation results obtained here suggest that there is a stronger apoptotic potential existing in the normal state of cancer cells, which is suppressed by the negative crosstalk AKT-ASK1 between PI3K/AKT and ASK1/p38 signaling pathways. The release of the apoptotic potential due to the fading away of negative crosstalk AKT-ASK1 upon EGFR inactivation could be an important factor causing the cell death, in addition to the inhibition of proliferation/pro-survival signaling. In particular, the rapid release of accumulated apoptotic potential could be a key reason that leads to the sensitivity of tumor cells to the killing effects of drugs that target the addicted oncogene.

### Experimental evidences for the existence of ROS and apoptotic potential in EGFR-addicted cancer cells

**Figure 7:**
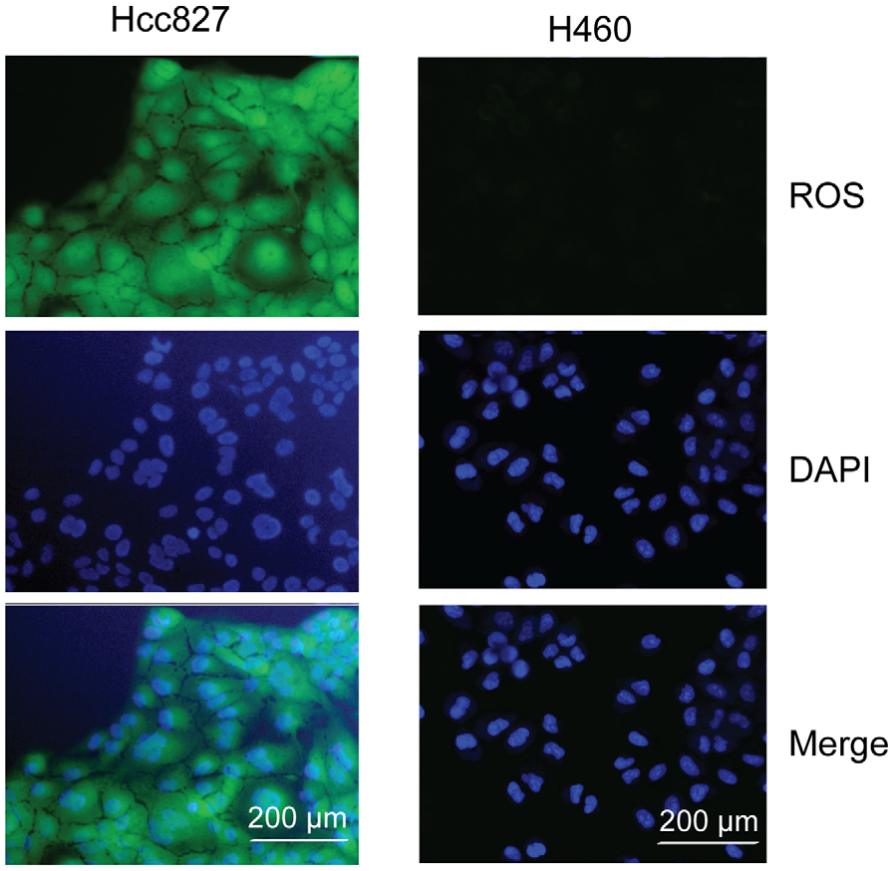
ROS levels in HCC827 and H460 cells. The ROS probe signals as well as the DAPI nuclear localization in HCC827 and H460 were presented alone or merged (Merge).

The model described above is somewhat artificial, which may not correspond closely with the actual EGFR addicted cancer cells. Therefore, we also performed a series of experiments to examine several key issues related to our modeling. Two non-small cell lung cancer (NSCLC) cell lines HCC827 and NCI-H460 (H460) were selected for the experiments; HCC827 is a typical EGFR addicted cancer cell line, and H460 is not EGFR addicted, which is for comparison. The first experiment was devised to investigate ROS levels in the two NSCLC cell lines. The intracellular ROS level was displayed by 2′,7′-dichlorodihydrofluorescein diacetate (DCFH-DA) [56], a ROS responsive molecular probe, and visualized by using an inverted fluorescence microscope. As shown in Figure 7, a very high level of ROS was detected in the EGFR-addicted HCC827 cells, whereas ROS could hardly be perceived in H460 cells. This finding reflects one fact that HCC827 suffers a large ROS stress, or in other words, HCC827 bears a stronger apoptotic potential. Even so HCC827 still keeps vigorous proliferation ability, suggesting that the apoptotic potential is well suppressed.

**Figure 8:**
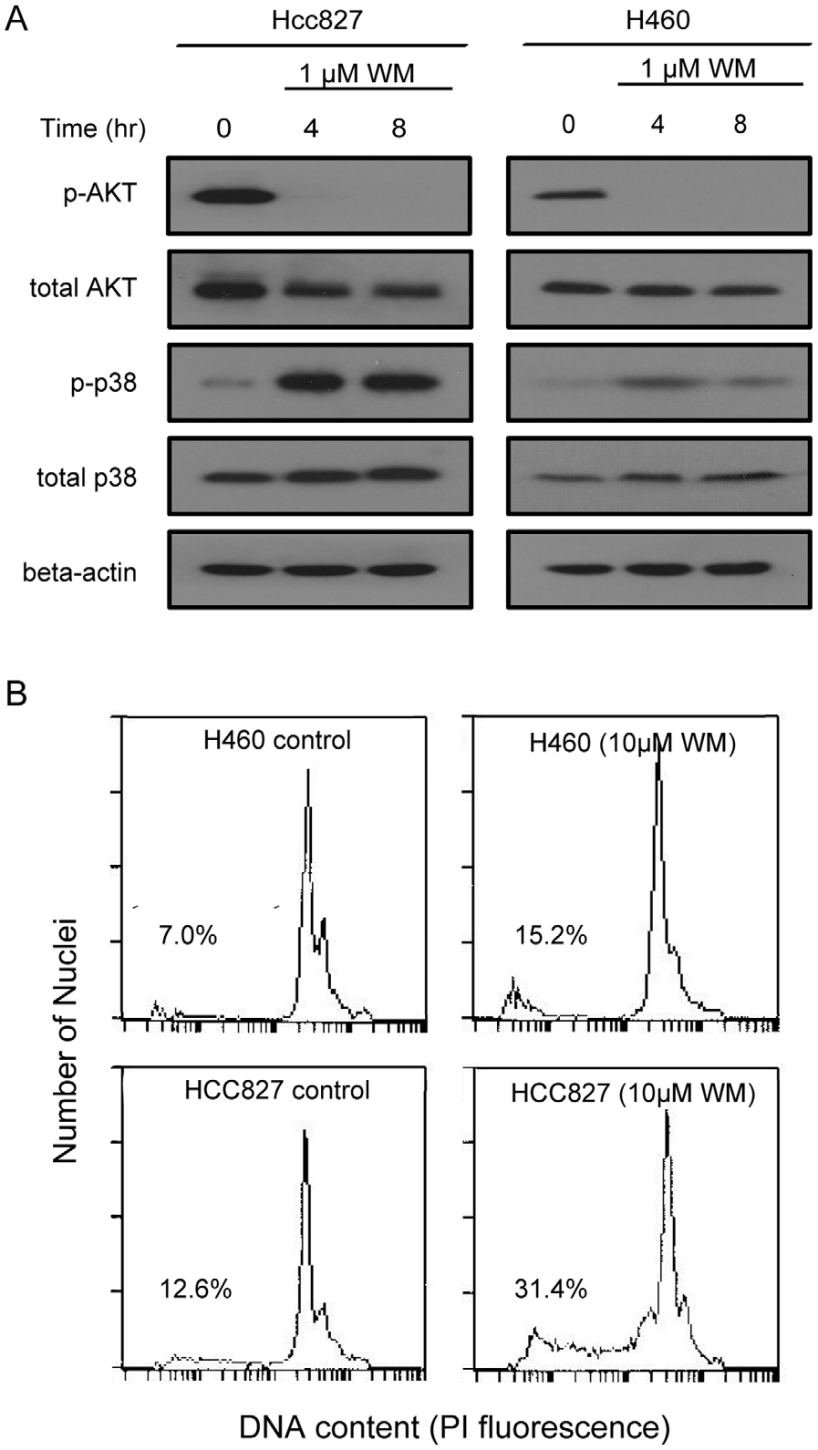
AKT inhibition induces differential p38 phosphorylation and apoptosis in HCC827 and H460. (A) Immunoblotting with indicated antibodies to show the phosphorylation of AKT and p38 in HCC827 and H460 cells treated with 1 µM wortmannin (WM). (B) Apoptosis of HCC827 and H460 cells with or without 10 µM wortmannin (WM) treatment for 24 hours. The apoptosis rates are given as percentages.

Subsequently western blot analysis and flow cytometry (FCM) assays were used to investigate whether the activation of p38 as well as the apoptosis of cancer cells can be triggered through AKT inhibition. We treated HCC827 and H460 cells with 1 µM wortmannin; wortmannin is an inhibitor of PI3K/AKT signaling. As shown in Figure 8A, wortmannin treatment of HCC827 cells can cause almost complete inhibition of AKT and large accumulation of p-p38 within 4 hours of treatment. For H460 cells, wortmannin treatment can also cause almost complete inhibition of the AKT, but very small accumulation of p-p38 without changing the intracellular ROS level (Figure S1). The results of FCM assays (Figure 8B) show that wortmannin treatment can result in increases of apoptosis rates both in HCC827 and H460 cells. In comparison with H460, HCC827 cells exhibited much more apoptosis after 10 µM wortmannin treatment. These experiments demonstrate that the inactivation of survival effector p-AKT leads to the rapid release of accumulated apoptotic potential in EGFR-addicted cancer cells, which may be an important reason that leads to the sensitivity of tumor cells to drugs that target the addicted oncogene EGFR.

**Figure 9:**
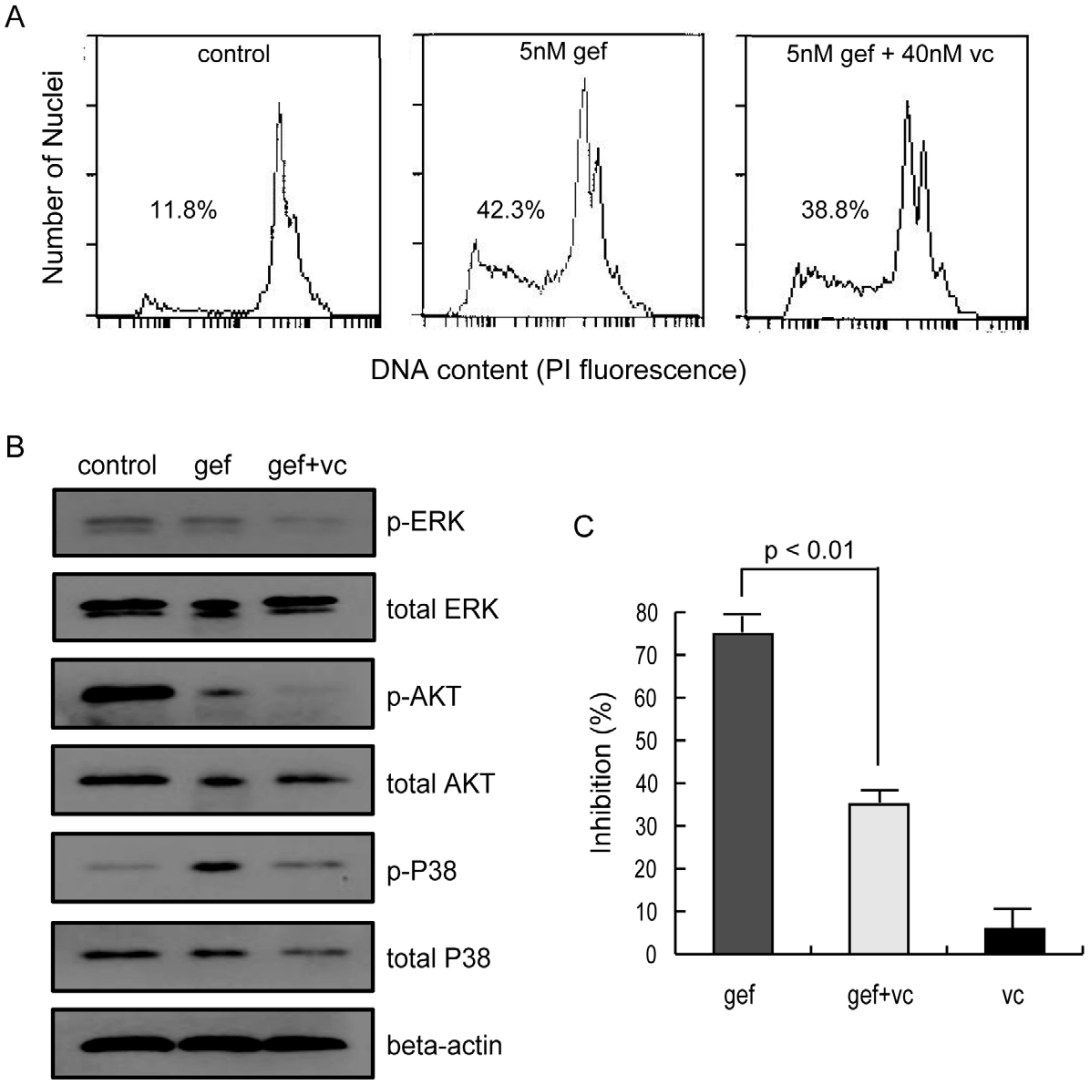
Vitamin c treatment reduces the apoptosis of HCC827 cells after gefitinib treatment. HCC827 cells were treated with vitamin c (vc), gefitinib (gef) or their combination (gef+vc). (A) Flow cytometry detecting the apoptosis of HCC827 cells under each treatment for 24 hours. The apoptosis rates of treatments are given as percentages. (B) Immunoblotting with indicated antibodies to show the phosphorylation of ERK, AKT and p38 in HCC827 cells treated by indicated agents. 1 µM gefitinib, 1 µM vitamin c or their combination were used. (C) The cell viability of HCC827 cells exposure to indicated agents measured by MTT. 1 nM gefitinib, 10 nM vitamin c or their combination were used.

Since we have demonstrated the existence of apoptotic potential due to ROS stress in EGFR-addicted cancer cells, we further postulate that the easement of ROS stress may desensitize the cancer cells to the targeted treatment. To test this hypothesis, we treated HCC827 cells with vitamin c, an antioxidant that can be used to partly mitigate the ROS stress. Then FCM and western blot assays were carried out to test the cell apoptosis exposure to the EGFR inhibitor gefitinib. As shown in Figure 9A, vitamin c can decrease the apoptosis of HCC827 cells treated by gefitinib compared with the control in which no vitamin c was used. Figure 9B depicts the results of western blot assays, which show that the mitigation of ROS indeed reduced the p-p38 level in HCC827 cells treated by gefitinib. At the same time, we also noticed that the alleviation of ROS decreased the p-ERK and p-AKT levels as well. A possible explanation could be that ROS stress can promote proliferation/survival, in addition to induce apoptosis [57]–[59], hence the mitigation of ROS consequentially leads to a decrease of p-ERK and p-AKT. We acknowledge that this did not get reflection from our modeling results since our mathematic model ignored the signaling from ROS to ERK/AKT, which is not known at present. In addition, a MTT assay was carried out to test the viability of HCC827 cells. The result shows that vitamin c can decrease the cytotoxicity of gefitinib in HCC827 cells (Figure 9C). These results suggest that the sensitivity of HCC827 cells to the EGFR inhibitor gefitinib is reduced due to the mitigation of apoptotic potential.

## Discussion

The concept of “oncogene addiction” has now been accepted by more and more researchers through the past decade. Nevertheless, the mechanism underlying the oncogene addiction is far from understood. In this investigation, we developed a mathematic model of EGFR-associated signaling network. We acknowledge that the established network model does not include all the signaling pathways and components that regulate the survival and apoptosis of cancer cells; establishment of a complete network model is impractical at present. From another point of view, ignoring other alternative signaling pathways may give a lucky fluke that the established model more closes to the EGFR addicted state.

Simulations with the validated EGFR-associated network model together with the experimental validation reveal the existence of apoptotic potential. The apoptotic potential could be induced by various stresses, typically for example, ROS. Cancer cells often show increased levels of intracellular ROS [60], [61]. Many factors may contribute to ROS generation [61]. The activation of oncogene EGFR can bring on ROS generation via the sequential activation of PI3K, Rac and NADPH oxidase [48]. In addition, hypoxia-reperfusion in the tumor microenvironment can lead to ROS production, which in turn can initiate a viscous cycle of mitochondrial damage and further ROS generation [62]. In normal cells, the ROS could be alleviated by engaging glycolysis and down-regulating mitochondrial function [63]. Cancer cells often have deficiency in the ROS reduction system due to genetic alterations, which hinder the clearance of ROS [61]. The accumulation of ROS usually results in the apoptosis through the sequential ROS/ASK1/p38 activation [47], [64]. Nevertheless, the activation of pro-apoptotic effector p38 is inhibited by negative crosstalk AKT-ASK1 in cancer cells [52], [65]. Once the mitigation of negative crosstalk AKT-ASK1, through for example, inactivation of AKT, the apoptotic potential can be released, hence inducing cell apoptosis [53].

The rapid release of accumulated apoptotic potential following oncogene inhibition is an important reason that leads to the sensitivity of tumor cells to the killing effects of drug that target the addicted oncogene, which is the most important hallmark of oncogene addiction. However, it does not mean that the overall apoptotic outcome in response to oncogene inactivation is contributed only by the apoptotic potential. It has been recognized that EGFR can produce pro-apoptotic outcome through activating some downstream effector pathways that have been linked to pro-apoptotic outcomes. For example, EGFR can bind directly to the so-called “death ligand” FAS/CD95 [66], hence leading to apoptotic outcome. The downstream Ras can be pro-apoptotic via an interaction with the effector target Nore1 [67].

Findings in this study may also have clinical implications in targeted cancer therapies as well as in the anti-cancer drug development. For example, sole use of ERK or AKT inhibitor may not bring a good therapeutic effect even to patients with EGFR-addicted cancers. Faber et al. [68] have demonstrated that PI3K/AKT inhibition did not promote substantial apoptosis in the EGFR addicted cancers. However, blockading both PI3K/AKT and Ras/MEK simultaneously led to apoptosis to similar levels as the EGFR inhibitors. In addition, the use of agents that benefit the increase of apoptotic potential may further increase the sensitivity of tumor cells to targeted drugs. For example, it has been reported that ROS-generating agents have selective killing effects in targeted-therapy-resistant cancers [69], [70]. Conversely, agents that can help to mitigate apoptotic potential could decrease the sensitivity of tumor cells to the EGFR inhibitors [59], which should be avoided to use clinically. Finally, this study also suggests that a good anti-cancer target should have a character that its functional inhibition should benefit the release of accumulated apoptosis potential in cancer cells, in addition to the blockade of survival.

In summary, our systems biology modeling reveals that there is a stronger apoptotic potential existing in the EGFR-addicted cancers, which is largely suppressed by the negative crosstalk between PI3K/AKT and ASK1/p38 signaling pathways. Inhibition of survival signals and concomitant release of accumulated apoptotic potential jointly contribute to the tumor cell death following the inhibition of addicted oncogene in EGFR addicted cancers. Overall, this is the first attempt to understand “oncogene addiction” from the systems biology's viewpoint. Further insights into the mechanisms underlying the oncogene addiction from the systems biology's viewpoint are still strongly required. Any advances in this field would ultimately benefit the targeted cancer therapy as well as the anti-cancer drug development.

## Materials and Methods

### Model Development

The network model was developed with the aid of Matlab (MathWorks, MA, USA, http://www.mathworks.com). The information for all the signaling pathways and topology of the network was collected from various published works. Molecular interactions in the model were described by a set of coupled ODEs, which were derived based on laws of Mass Action. For enzymatic reactions, the reaction constant (K) and turn-over rates (Kcats) were used instead of the Michaelis-Menten constants (Km) that are primarily applicable to steady-state models. These ODEs were solved using the sundials solvers in MatLab.

### Sensitivity Analysis

Sensitivity analysis was performed to identify important species that control signaling networks. Normalized, time-integrated sensitivities of each species were calculated by varying the initial value of them and simulating the perturbed system output (for example, phospho-AKT, -ERK and -p38). The normalized sensitivity S was calculated according to the following equation:
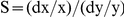
Where S corresponds to the normalized integrated sensitivity and x is any output species and y is each species with non-zero initial amount.

### Cell Lines and Cell Culture

The human lung cancer cell lines NCI-HCC827 and NCI-H460 were obtained from the American Type Culture Collection (ATCC, Manassas, VA), and were cultured in RPMI 1640 (Life Technologies, Bedford, MA) containing 10% heat-inactivated fetal bovine serum, 100 units/ml penicillin and 100 units/ml streptomycin in a humid chamber at 37°C under 5% CO_2_ in atmosphere. Gefitinib (AstraZeneca), wortmannin (Cell Signaling) and vitamin C (Sigma) were used as drugs treating cancer cells. Stock solutions of all drugs were prepared in dimethyl sulfoxide (DMSO) and stored at −20°C.

### Flow cytometry assays

Flow cytometric analysis was done to identify apoptotic cells and to measure the percentage of apoptotic cells after propidium iodide staining in hypotonic buffer as described [69]. Briefly, cells were suspended in 1 mL hypotonic fluorochrome solution containing 50 µg propidium iodide/mL in 0.1% sodium citrate plus 0.1% Triton X-100 and the cells were analyzed by flow cytometer (ESP Elite, Beckman-Coulter, Miami, FL). Apoptotic cells appeared in the cell cycle distribution as cells with a DNA content of less than that of G1 cells and were estimated with Listmode software.

### Cell viability assay

NCI-HCC827 and NCI-H460 cells (4–5×10^3^) were seeded in 96-well plates and treated with gefitinib or vitamin c, or combination of them. Followed by 3 day incubation after these drug treatments, cells were then incubated with 3-(4,5-Dimethylthiazol-2-yl)-2,5-diphenyltetrazolium bromide (MTT) reagent for 4 hours, lysed with DMSO, and quantified by a plate reader. The data were graphically displayed using GraphPad Prism version 5.00 for Windows. Each point (mean (+/− standard deviation)) represents growth of treated cells compared to untreated cells. The curves were fitted using a non-linear regression model with a sigmoidal dose response.

### Western Blot Analysis

Whole-cell lysates were extracted with cell lysis buffer for Western blotting (Beyotime) supplemented with 1 mM PMSF (Beyotime). Protein content was determined using BCA protein assay kit (Beyotime). Lysates were mixed with 5×sodium dodecyl sulfate (SDS)- polyacrylamide gel electrophoresis (PAGE) sample loading buffer (Beyotime) and denatured in a boiling water bath for 5 minutes. Protein extracts were separated by SDS-PAGE on 10% polyacrylamide Tris/glycine gels and transferred onto a polyvinylidene difluoride membrane (Millipore). Membranes were blocked in Tris-buffered saline (TBS) containing 0.1% Tween and 10% nonfat dry milk for 1 hour. Antibodies were diluted in TBS containing 0.1% Tween and 5% nonfat dry milk. Blots were incubated with the corresponding primary antibody (1∶1000) overnight. Then, blots were incubated for 1 hour with the corresponding horseradish peroxidase-linked secondary antibodies (Santa Cruz) diluted 1∶5,000 in TBS containing 0.05% Tween, and 0.5% nonfat dry milk. The primary antibodies used for the Western blot analyses include anti-p38α MAPK, anti-phospho-p38α MAPK, anti-ERK 1/2, anti-phospho-ERK1/2, anti-AKT, and anti-phospho-AKT from Cell Signaling Technology; and anti-β actin from Sigma. Following secondary antibody reaction, the proteins were detected with BeyoECL reagents (Beyotime) by exposure on a Kodak film.

### Determination of Intracellular ROS Levels

To visualize intracellular ROS of cancer cells, proliferating cells were grown on 6 well plates, washed once with warm PBS, and incubated with 10 mM 2′,7′-dichlorodihydrofluorescein diacetate (DCFH-DA, Beyotime) in warm PBS supplemented with 5.5 mM glucose. After 10 minutes at 37°C, PBS was replaced with complete culture medium, and cells were incubated for an additional 20–30 minutes, washed once again with warm PBS. Cells were incubated with 1 mM 4,6-diamidino-2-phenylindole (DAPI) for nuclei staining (Sigma) and intracellular ROS levels were visualized by using an inverted fluorescence microscope, Leica DM5000B, coupled to a Leica DC500 camera. Pictures were taken at 20× magnification with the Leica IM50 software.

### Statistical analysis

Data were presented as the mean±SD error of the mean. Student's t test was used for comparison among different groups. A P value of less than 0.05 is considered statistically significant.

## Supporting Information

Figure S1 **ROS levels in H460 cells with or without gefitinib treatment.** The ROS probe signals as well as the DAPI nuclear localization in H460 cells were presented alone or merged (merge).
(TIF)

Table S1 Biochemical reactions involved in the computational model together with corresponding parameters.
(DOC)

Table S2 Initial conditions of the computational model.
(DOC)
