# Supplementary figures and images for: Systems Biology Modeling Reveals a Possible Mechanism of the Tumor Cell Death upon Oncogene Inactivation in EGFR Addicted Cancers
Source: PLoS One. 2011 Dec 14;6(12):e28930. doi: 10.1371/journal.pone.0028930 (PMC3237568; doi:10.1371/journal.pone.0028930)

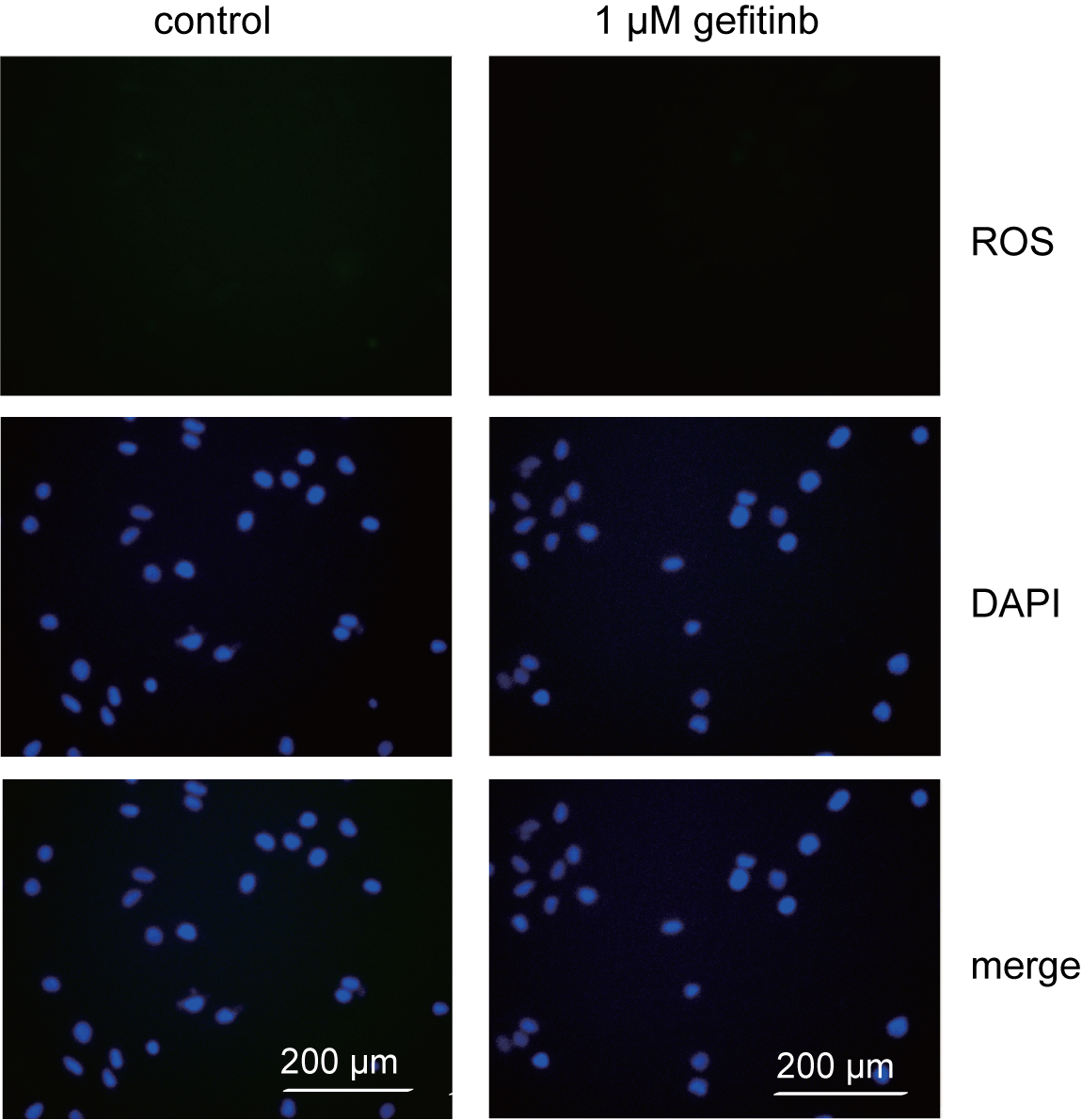

Supplement: Figure S1 — ROS levels in H460 cells with or without gefitinib treatment. The ROS probe signals as well as the DAPI nuclear localization in H460 cells were presented alone or merged (merge). (TIF) [file pone.0028930.s001.tif]
