# Supplementary material for: Systems Biology Modeling Reveals a Possible Mechanism of the Tumor Cell Death upon Oncogene Inactivation in EGFR Addicted Cancers
Source: PLoS One. 2011 Dec 14;6(12):e28930. doi: 10.1371/journal.pone.0028930 (PMC3237568; doi:10.1371/journal.pone.0028930)
Supplement: Table S1 — Biochemical reactions involved in the computational model together with corresponding parameters. (DOC) [file pone.0028930.s002.doc]

**Table S1. Biochemical reactions involved in the computational model with the corresponding parameters.**

| **No.** | **Reaction/process** | **Kinetic constants** | |
| --- | --- | --- | --- |
|  |  | **kf (nM^-1^×s^-1^ or s^-1^)** | **kr (nM)** |
| **Binding of EGF and EGFR** | | | |
| 1 | EGF + EGFR = [EGF-EGFR] | 0.0001 | 0.175 |
| **Dimerization of EGF-EGFR** | | | |
| 2 | [EGF-EGFR] + [EGF-EGFR] = [(EGF-EGFR)2] | 0.5005 | 0.1717 |
| **Phosphorylation and dephosphorylation of EGFR** | | | |
| 3 | [(EGF-EGFR)2] -> [(EGF-EGFR)2_p] | 0.6496 |  |
| 4 | [(EGF-EGFR)2_p] + RTKpase = [(EGF-EGFR)2_p-RTKpase] | 0.0464 | 3.0048 |
| 5 | [(EGF-EGFR)2_p-RTKpase] -> [(EGF-EGFR)2] + RTKpase | 29.8531 |  |
| **Recruitment of SOS** | | | |
| 6 | [(EGF-EGFR)2_p] + Grb2 = [(EGF-EGFR)2_p-Grb2] | 0.0388 | 0.5737 |
| 7 | [(EGF-EGFR)2_p] + Shc = [(EGF-EGFR)2_p-Shc] | 0.0392 | 4.6874 |
| 8 | [(EGF-EGFR)2_p-Shc] = [(EGF-EGFR)2_p-Shc_p] | 5.78242 | 0.2661 |
| 9 | [(EGF-EGFR)2_p-Shc_p] + Grb2 = [(EGF-EGFR)2_p-Shc_p-Grb2] | 0.0023 | 3.5195 |
| 10 | [(EGF-EGFR)2_p-Grb2] + SOS = [(EGF-EGFR)2_p-Grb2-SOS] | 0.0191 | 3.1051 |
| 11 | [(EGF-EGFR)2_p-Shc_p-Grb2] + SOS = [(EGF-EGFR)2_p-Shc_p-Grb2-SOS] | 0.0191 | 3.1051 |
| 12 | [(EGF-EGFR)2_p] + [Grb2-SOS] = [(EGF-EGFR)2_p-Grb2-SOS] | 0.0027 | 0.025 |
| 13 | [(EGF-EGFR)2_p-Shc_p] + [Grb2-SOS] = [(EGF-EGFR)2_p-Shc_p-Grb2-SOS] | 0.01 | 0.045 |
| 14 | SOS + Grb2 = [Grb2-SOS] | 0.0001 | 0.0015 |
| 15 | Shc_p -> Shc | 0.2661 |  |
| 16 | [(EGF-EGFR)2_p] + Shc_p = [(EGF-EGFR)2_p-Shc_p] | 0.0003 | 4.481 |
| **Activation of RasGDP** | | | |
| 17 | RasGDP + [(EGF-EGFR)2_p-Grb2-SOS] = [(EGF-EGFR)2_p-Grb2-SOS-RasGDP] | 10 | 0.18 |
| 18 | [(EGF-EGFR)2_p-Grb2-SOS-RasGDP] -> [(EGF-EGFR)2_p-Grb2-SOS] + RasGTP | 0.1434 |  |
| 19 | RasGDP + [(EGF-EGFR)2_p-Shc_p-Grb2-SOS] = [(EGF-EGFR)2_p-Shc_p-Grb2-SOS-RasGDP] | 10 | 0.18 |
| 20 | [(EGF-EGFR)2_p-Shc_p-Grb2-SOS-RasGDP] -> [(EGF-EGFR)2_p-Shc_p-Grb2-SOS] + RasGTP | 0.1434 |  |
| **Activation and deactivation of Raf** | | | |
| 21 | Raf + RasGTP = [Raf-RasGTP] | 0.5 | 0.05 |
| 22 | [Raf-RasGTP] -> Raf_p + RasGTP | 10 |  |
| 23 | Raf_p + Pase1 = [Raf_p-Pase1] | 0.0717 | 0.2 |
| 24 | [Raf_p-Pase1] -> Raf + Pase1 | 1 |  |
| **Activation and deactivation of ERK** | | | |
| 25 | MEK + Raf_p = [MEK-Raf_p] | 0.004 | 0.01833 |
| 26 | [MEK-Raf_p] -> MEK_p + Raf_p | 3.5 |  |
| 27 | MEK_p + Raf_p = [MEK_p-Raf_p] | 0.004 | 0.01833 |
| 28 | [MEK_p-Raf_p] -> MEK_pp + Raf_p | 2.9 |  |
| 29 | MEK_pp + Pase2 = [MEK_pp-Pase2] | 0.0143 | 0.8 |
| 30 | [MEK_pp-Pase2] -> MEK_p + Pase2 | 0.058 |  |
| 31 | MEK_p + Pase2 = [MEK_p-Pase2] | 0.00025 | 0.5 |
| 32 | [MEK_p-Pase2] -> MEK + Pase2 | 0.058 |  |
| 33 | ERK + MEK_pp = [ERK-MEK_pp] | 0.1997 | 100 |
| 34 | [ERK-MEK_pp] -> ERK_p + MEK_pp | 20 |  |
| 35 | ERK_p + MEK_pp = [ERK_p-MEK_pp] | 0.1997 | 100 |
| 36 | [ERK_p-MEK_pp] -> ERK_pp + MEK_pp | 0.2004 |  |
| 37 | ERK_pp + Pase3 = [ERK_pp-Pase3] | 0.2003 | 100 |
| 38 | [ERK_pp-Pase3] -> ERK_p + Pase3 | 0.9966 |  |
| 39 | ERK_p + Pase3 = [ERK_p-Pase3] | 0.1993 | 100 |
| 40 | [ERK_p-Pase3] -> ERK + Pase3 | 19.9851 |  |
| **Recruitment and phosphorylation of Gab1** | | | |
| 41 | [(EGF-EGFR)2_p-Grb2] + Gab1 = [(EGF-EGFR)2_p-Grb2-Gab1] | 0.0051 | 7.0487 |
| 42 | [(EGF-EGFR)2_p-Grb2] + Gab1_p = [(EGF-EGFR)2_p-Grb2-Gab1_p] | 0.0051 | 7.0487 |
| 43 | [(EGF-EGFR)2_p-Shc_p-Grb2] + Gab1 = [(EGF-EGFR)2_p-Shc_p-Grb2-Gab1] | 0.0051 | 7.0487 |
| 44 | [(EGF-EGFR)2_p-Shc_p-Grb2] + Gab1_p = [(EGF-EGFR)2_p-Shc_p-Grb2-Gab1_p] | 0.0051 | 7.0487 |
| 45 | [(EGF-EGFR)2_p-Grb2-Gab1] = [(EGF-EGFR)2_p-Grb2-Gab1_p] | 6.44315 | 0.157405 |
| 46 | [(EGF-EGFR)2_p-Shc_p-Grb2-Gab1] = [(EGF-EGFR)2_p-Shc_p-Grb2-Gab1_p] | 6.44315 | 0.157405 |
| 47 | [(EGF-EGFR)2_p] + [Grb2-Gab1] = [(EGF-EGFR)2_p-Grb2-Gab1] | 0.0027 | 0.025 |
| 48 | [(EGF-EGFR)2_p] + [Grb2-Gab1_p] = [(EGF-EGFR)2_p-Grb2-Gab1_p] | 0.0027 | 0.025 |
| 49 | [(EGF-EGFR)2_p-Shc_p] + [Grb2-Gab1] = [(EGF-EGFR)2_p-Shc_p-Grb2-Gab1] | 0.01 | 0.045 |
| 50 | [(EGF-EGFR)2_p-Shc_p] + [Grb2-Gab1_p] = [(EGF-EGFR)2_p-Shc_p-Grb2-Gab1_p] | 0.01 | 0.045 |
| 51 | Gab1_p + Grb2 = [Grb2-Gab1_p] | 0.000610564 | 6.28346 |
| 52 | Gab1 + Grb2 = [Grb2-Gab1] | 0.000610564 | 6.28346 |
| 53 | Gab1_p -> Gab1 | 0.01 |  |
| **Recruitment of RasGAP** | | | |
| 54 | [(EGF-EGFR)2_p] + RasGAP = [(EGF-EGFR)2_p-RasGAP] | 0.0056 | 3.9967 |
| 55 | [(EGF-EGFR)2_p-Grb2-Gab1_p] + RasGAP = [(EGF-EGFR)2_p-Grb2-Gab1_p-RasGAP] | 0.0056 | 3.9967 |
| 56 | [(EGF-EGFR)2_p-Shc_p-Grb2-Gab1_p] + RasGAP = [(EGF-EGFR)2_p-Shc_p-Grb2-Gab1_p-RasGAP] | 0.0056 | 3.9967 |
| **RasGTP deactivation** | | | |
| 57 | RasGTP -> RasGDP | 1 |  |
| 58 | [(EGF-EGFR)2_p-Grb2-Gab1_p-RasGAP] + RasGTP = [(EGF-EGFR)2_p-Grb2-Gab1_p-RasGAP-RasGTP] | 0.02854 | 0.96 |
| 59 | [(EGF-EGFR)2_p-Grb2-Gab1_p-RasGAP-RasGTP] -> [(EGF-EGFR)2_p-Grb2-Gab1_p-RasGAP] + RasGDP | 7.76 |  |
| 60 | [(EGF-EGFR)2_p-Shc_p-Grb2-Gab1_p-RasGAP] + RasGTP = [(EGF-EGFR)2_p-Shc_p-Grb2-Gab1_p-RasGAP-RasGTP] | 0.02854 | 0.96 |
| 61 | [(EGF-EGFR)2_p-Shc_p-Grb2-Gab1_p-RasGAP-RasGTP] -> [(EGF-EGFR)2_p-Shc_p-Grb2-Gab1_p-RasGAP] + RasGDP | 7.76 |  |
| 62 | [(EGF-EGFR)2_p-RasGAP] + RasGTP = [(EGF-EGFR)2_p-RasGAP-RasGTP] | 0.02854 | 0.96 |
| 63 | [(EGF-EGFR)2_p-RasGAP-RasGTP] -> [(EGF-EGFR)2_p-RasGAP] + RasGDP | 7.76 |  |
| **PI3K binding and PIP2 activation** | | | |
| 64 | [(EGF-EGFR)2_p-Grb2-Gab1_p] + PI3K = [(EGF-EGFR)2_p-Grb2-Gab1_p-PI3K] | 0.0366 | 0.01 |
| 65 | [(EGF-EGFR)2_p-Shc_p-Grb2-Gab1_p] + PI3K = [(EGF-EGFR)2_p-Shc_p-Grb2-Gab1_p-PI3K] | 0.0366 | 0.01 |
| 66 | [(EGF-EGFR)2_p-Grb2-Gab1_p-PI3K] + PIP2 = [(EGF-EGFR)2_p-Grb2-Gab1_p-PI3K-PIP2] | 2 | 3.5 |
| 67 | [(EGF-EGFR)2_p-Grb2-Gab1_p-PI3K-PIP2] -> [(EGF-EGFR)2_p-Grb2-Gab1_p-PI3K] + PIP3 | 100 |  |
| 68 | [(EGF-EGFR)2_p-Shc_p-Grb2-Gab1_p-PI3K] + PIP2 = [(EGF-EGFR)2_p-Shc_p-Grb2-Gab1_p-PI3K-PIP2] | 2 | 3.5 |
| 69 | [(EGF-EGFR)2_p-Shc_p-Grb2-Gab1_p-PI3K-PIP2] -> [(EGF-EGFR)2_p-Shc_p-Grb2-Gab1_p-PI3K] + PIP3 | 100 |  |
| **Membrane complex internalization** | | | |
| 70 | [(EGF-EGFR)2] = [i(EGF-EGFR)2] | 0.8 | 0.005 |
| 71 | [(EGF-EGFR)2_p] = [i(EGF-EGFR)2_p] | 0.8 | 0.005 |
| 72 | [(EGF-EGFR)2_p-RTKpase] = [i(EGF-EGFR)2_p-RTKpase] | 0.8 | 0.005 |
| 73 | [(EGF-EGFR)2_p-Shc] = [i(EGF-EGFR)2_p-Shc] | 0.8 | 0.005 |
| 74 | [(EGF-EGFR)2_p-Shc_p] = [i(EGF-EGFR)2_p-Shc_p] | 0.8 | 0.005 |
| 75 | [(EGF-EGFR)2_p-Grb2] = [i(EGF-EGFR)2_p-Grb2] | 0.8 | 0.005 |
| 76 | [(EGF-EGFR)2_p-Shc_p-Grb2] = [i(EGF-EGFR)2_p-Shc_p-Grb2] | 0.8 | 0.005 |
| 77 | [(EGF-EGFR)2_p-Shc_p-Grb2-SOS] = [i(EGF-EGFR)2_p-Shc_p-Grb2-SOS] | 0.8 | 0.005 |
| 78 | [(EGF-EGFR)2_p-Grb2-SOS] = [i(EGF-EGFR)2_p-Grb2-SOS] | 0.8 | 0.005 |
| 79 | [(EGF-EGFR)2_p-Grb2-Gab1] = [i(EGF-EGFR)2_p-Grb2-Gab1] | 0.8 | 0.005 |
| 80 | [(EGF-EGFR)2_p-Shc_p-Grb2-Gab1] = [i(EGF-EGFR)2_p-Shc_p-Grb2-Gab1] | 0.8 | 0.005 |
| 81 | [(EGF-EGFR)2_p-Grb2-Gab1_p] = [i(EGF-EGFR)2_p-Grb2-Gab1_p] | 0.8 | 0.005 |
| 82 | [(EGF-EGFR)2_p-Shc_p-Grb2-Gab1_p] = [i(EGF-EGFR)2_p-Shc_p-Grb2-Gab1_p] | 0.8 | 0.005 |
| 83 | [(EGF-EGFR)2_p-RasGAP] = [i(EGF-EGFR)2_p-RasGAP] | 0.8 | 0.005 |
| 84 | [(EGF-EGFR)2_p-Grb2-Gab1_p-RasGAP] = [i(EGF-EGFR)2_p-Grb2-Gab1_p-RasGAP] | 0.8 | 0.005 |
| 85 | [(EGF-EGFR)2_p-Shc_p-Grb2-Gab1_p-RasGAP] = [i(EGF-EGFR)2_p-Shc_p-Grb2-Gab1_p-RasGAP] | 0.8 | 0.005 |
| 86 | [(EGF-EGFR)2_p-Grb2-Gab1_p-PI3K] = [i(EGF-EGFR)2_p-Grb2-Gab1_p-PI3K] | 0.8 | 0.005 |
| 87 | [(EGF-EGFR)2_p-Shc_p-Grb2-Gab1_p-PI3K] = [i(EGF-EGFR)2_p-Shc_p-Grb2-Gab1_p-PI3K] | 0.8 | 0.005 |
| **Internalized dimerization of EGFR** | | | |
| 88 | [iEGF-EGFR] + [iEGF-EGFR] = [i(EGF-EGFR)2] | 0.5005 | 0.1717 |
| **Internalized phosphorylation and dephosphorylation of EGFR** | | | |
| 89 | [i(EGF-EGFR)2] -> [i(EGF-EGFR)2_p] | 0.6496 |  |
| 90 | [i(EGF-EGFR)2_p] + RTKpase = [i(EGF-EGFR)2_p-RTKpase] | 0.0464 | 3.0048 |
| 91 | [i(EGF-EGFR)2_p-RTKpase] -> [i(EGF-EGFR)2] + RTKpase | 29.8531 |  |
| **Internalized PI3K binding** | | | |
| 92 | [i(EGF-EGFR)2_p-Grb2-Gab1_p] + PI3K = [i(EGF-EGFR)2_p-Grb2-Gab1_p-PI3K] | 0.0366 | 0.01 |
| 93 | [i(EGF-EGFR)2_p-Shc_p-Grb2-Gab1_p] + PI3K = [i(EGF-EGFR)2_p-Shc_p-Grb2-Gab1_p-PI3K] | 0.0366 | 0.01 |
| **Internalized recruitment of SOS** | | | |
| 94 | [i(EGF-EGFR)2_p] + Grb2 = [i(EGF-EGFR)2_p-Grb2] | 0.0388 | 0.5737 |
| 95 | [i(EGF-EGFR)2_p] + Shc = [i(EGF-EGFR)2_p-Shc] | 0.0392 | 4.6874 |
| 96 | [i(EGF-EGFR)2_p-Shc] = [i(EGF-EGFR)2_p-Shc_p] | 5.78242 | 0.2661 |
| 97 | [i(EGF-EGFR)2_p-Shc_p] + Grb2 = [i(EGF-EGFR)2_p-Shc_p-Grb2] | 0.0023 | 3.5195 |
| 98 | [i(EGF-EGFR)2_p-Grb2] + SOS = [i(EGF-EGFR)2_p-Grb2-SOS] | 0.0191 | 3.1051 |
| 99 | [i(EGF-EGFR)2_p-Shc_p-Grb2] + SOS = [i(EGF-EGFR)2_p-Shc_p-Grb2-SOS] | 0.0191 | 3.1051 |
| 100 | [i(EGF-EGFR)2_p] + [Grb2-SOS] = [i(EGF-EGFR)2_p-Grb2-SOS] | 0.0027 | 0.025 |
| 101 | [i(EGF-EGFR)2_p-Shc_p] + [Grb2-SOS] = [i(EGF-EGFR)2_p-Shc_p-Grb2-SOS] | 0.01 | 0.045 |
| 102 | [i(EGF-EGFR)2_p] + Shc_p = [i(EGF-EGFR)2_p-Shc_p] | 0.0003 | 4.481 |
| **Internalized activation of RasGDP** | | | |
| 103 | RasGDP + [i(EGF-EGFR)2_p-Grb2-SOS] = [i(EGF-EGFR)2_p-Grb2-SOS-RasGDP] | 10 | 0.18 |
| 104 | [i(EGF-EGFR)2_p-Grb2-SOS-RasGDP] -> [i(EGF-EGFR)2_p-Grb2-SOS] + RasGTP | 0.1434 |  |
| 105 | RasGDP + [i(EGF-EGFR)2_p-Shc_p-Grb2-SOS] = [i(EGF-EGFR)2_p-Shc_p-Grb2-SOS-RasGDP] | 10 | 0.18 |
| 106 | [i(EGF-EGFR)2_p-Shc_p-Grb2-SOS-RasGDP] -> [i(EGF-EGFR)2_p-Shc_p-Grb2-SOS] + RasGTP | 0.1434 |  |
| **Internalized recruitment and phosphorylation of Gab1** | | | |
| 107 | [i(EGF-EGFR)2_p-Grb2] + Gab1 = [i(EGF-EGFR)2_p-Grb2-Gab1] | 0.0051 | 7.0487 |
| 108 | [i(EGF-EGFR)2_p-Grb2] + Gab1_p = [i(EGF-EGFR)2_p-Grb2-Gab1_p] | 0.0051 | 7.0487 |
| 109 | [i(EGF-EGFR)2_p-Shc_p-Grb2] + Gab1 = [i(EGF-EGFR)2_p-Shc_p-Grb2-Gab1] | 0.0051 | 7.0487 |
| 110 | [i(EGF-EGFR)2_p-Shc_p-Grb2] + Gab1_p = [i(EGF-EGFR)2_p-Shc_p-Grb2-Gab1_p] | 0.0051 | 7.0487 |
| 111 | [i(EGF-EGFR)2_p-Grb2-Gab1] = [i(EGF-EGFR)2_p-Grb2-Gab1_p] | 6.44315 | 0.157405 |
| 112 | [i(EGF-EGFR)2_p-Shc_p-Grb2-Gab1] = [i(EGF-EGFR)2_p-Shc_p-Grb2-Gab1_p] | 6.44315 | 0.157405 |
| 113 | [i(EGF-EGFR)2_p] + [Grb2-Gab1] = [i(EGF-EGFR)2_p-Grb2-Gab1] | 0.0027 | 0.025 |
| 114 | [i(EGF-EGFR)2_p] + [Grb2-Gab1_p] = [i(EGF-EGFR)2_p-Grb2-Gab1_p] | 0.0027 | 0.025 |
| 115 | [i(EGF-EGFR)2_p-Shc_p] + [Grb2-Gab1] = [i(EGF-EGFR)2_p-Shc_p-Grb2-Gab1] | 0.01 | 0.045 |
| 116 | [i(EGF-EGFR)2_p-Shc_p] + [Grb2-Gab1_p] = [i(EGF-EGFR)2_p-Shc_p-Grb2-Gab1_p] | 0.01 | 0.045 |
| **Internalized recruitment of RasGAP** | | | |
| 117 | [i(EGF-EGFR)2_p] + RasGAP = [i(EGF-EGFR)2_p-RasGAP] | 0.0056 | 3.9967 |
| 118 | [i(EGF-EGFR)2_p-Grb2-Gab1_p] + RasGAP = [i(EGF-EGFR)2_p-Grb2-Gab1_p-RasGAP] | 0.0056 | 3.9967 |
| 119 | [i(EGF-EGFR)2_p-Shc_p-Grb2-Gab1_p] + RasGAP = [i(EGF-EGFR)2_p-Shc_p-Grb2-Gab1_p-RasGAP] | 0.0056 | 3.9967 |
| **Internalized deactivation of RasGTP** | | | |
| 120 | [i(EGF-EGFR)2_p-Grb2-Gab1_p-RasGAP] + RasGTP = [i(EGF-EGFR)2_p-Grb2-Gab1_p-RasGAP-RasGTP] | 0.02854 | 0.96 |
| 121 | [i(EGF-EGFR)2_p-Grb2-Gab1_p-RasGAP-RasGTP] -> [i(EGF-EGFR)2_p-Grb2-Gab1_p-RasGAP] + RasGDP | 7.76 |  |
| 122 | [i(EGF-EGFR)2_p-Shc_p-Grb2-Gab1_p-RasGAP] + RasGTP = [i(EGF-EGFR)2_p-Shc_p-Grb2-Gab1_p-RasGAP-RasGTP] | 0.02854 | 0.96 |
| 123 | [i(EGF-EGFR)2_p-Shc_p-Grb2-Gab1_p-RasGAP-RasGTP] -> [i(EGF-EGFR)2_p-Shc_p-Grb2-Gab1_p-RasGAP] + RasGDP | 7.76 |  |
| 124 | [i(EGF-EGFR)2_p-RasGAP] + RasGTP = [i(EGF-EGFR)2_p-RasGAP-RasGTP] | 0.02854 | 0.96 |
| 125 | [i(EGF-EGFR)2_p-RasGAP-RasGTP] -> [i(EGF-EGFR)2_p-RasGAP] + RasGDP | 7.76 |  |
| **AKT activation** | | | |
| 126 | PIP3 + PTEN = [PIP3-PTEN] | 0.003 | 50 |
| 127 | [PIP3-PTEN] -> PIP2 + PTEN | 3 |  |
| 128 | PIP3 + AKT = [PIP3-AKT] | 0.003 | 1 |
| 129 | [PIP3-AKT] + PDK1 = [PIP3-AKT-PDK1] | 0.003 | 1 |
| 130 | [PIP3-AKT-PDK1] -> [PIP3-AKT_p] + PDK1 | 3 |  |
| 131 | [AKT_pp-PIP3] -> PIP3 + AKT_pp | 0.2 |  |
| 132 | PIP3 + AKT_p = [PIP3-AKT_p] | 0.003 | 1 |
| 133 | [PIP3-AKT_p] + PDK1 = [PIP3-AKT_p-PDK1] | 0.003 | 1 |
| 134 | [PIP3-AKT_p-PDK1] -> [AKT_pp-PIP3] + PDK1 | 3 |  |
| 135 | AKT_pp + PP2A = [AKT_pp-PP2A] | 0.001 | 0.1 |
| 136 | [AKT_pp-PP2A] -> AKT_p + PP2A | 1.5 |  |
| 137 | AKT_p + PP2A = [AKT_p-PP2A] | 0.001 | 0.1 |
| 138 | [AKT_p-PP2A] -> AKT + PP2A | 1.5 |  |
| **Activated AKT suppresses Raf activity** | |  |  |
| 139 | Raf_p + AKT_pp = [AKT_pp-Raf_p] | 0.003 | 0.5 |
| 140 | [AKT_pp-Raf_p] -> AKT_pp + Raf_pt | 3 |  |
| 141 | Raf_pt -> Raf_p | 0.01 |  |
| **Activated ERK feedback** | | | |
| 142 | [(EGF-EGFR)2_p-Grb2-SOS] + ERK_pp = [(EGF-EGFR)2_p-Grb2-SOS-ERK_pp] | 0.5 | 1 |
| 143 | [(EGF-EGFR)2_p-Grb2-SOS-ERK_pp] -> Grb2 + SOS_pt + ERK_pp + [(EGF-EGFR)2_pt] | 0.0426 |  |
| 144 | [(EGF-EGFR)2_p-Shc_p-Grb2-SOS] + ERK_pp = [(EGF-EGFR)2_p-Shc_p-Grb2-SOS-ERK_pp] | 0.5 | 1 |
| 145 | [(EGF-EGFR)2_p-Shc_p-Grb2-SOS-ERK_pp] -> [Shc_p-Grb2] + SOS_pt + ERK_pp + [(EGF-EGFR)2_pt] | 0.0426 |  |
| 146 | [(EGF-EGFR)2_p-Grb2-Gab1] + ERK_pp = [(EGF-EGFR)2_p-Grb2-Gab1-ERK_pp] | 0.000185 | 1 |
| 147 | [(EGF-EGFR)2_p-Grb2-Gab1-ERK_pp] -> Grb2 + Gab1_pt + ERK_pp + [(EGF-EGFR)2_pt] | 0.2256 |  |
| 148 | [(EGF-EGFR)2_p-Shc_p-Grb2-Gab1] + ERK_pp = [(EGF-EGFR)2_p-Shc_p-Grb2-Gab1-ERK_pp] | 0.000185 | 1 |
| 149 | [(EGF-EGFR)2_p-Shc_p-Grb2-Gab1-ERK_pp] -> [Shc_p-Grb2] + Gab1_pt + ERK_pp + [(EGF-EGFR)2_pt] | 0.2256 |  |
| 150 | [(EGF-EGFR)2_p-Grb2-Gab1_p] + ERK_pp = [(EGF-EGFR)2_p-Grb2-Gab1_p-ERK_pp] | 0.000185 | 1 |
| 151 | [(EGF-EGFR)2_p-Grb2-Gab1_p-ERK_pp] -> Grb2 + Gab1_pt + ERK_pp + [(EGF-EGFR)2_pt] | 0.2256 |  |
| 152 | [(EGF-EGFR)2_p-Shc_p-Grb2-Gab1_p] + ERK_pp = [(EGF-EGFR)2_p-Shc_p-Grb2-Gab1_p-ERK_pp] | 0.000185 | 1 |
| 153 | [(EGF-EGFR)2_p-Shc_p-Grb2-Gab1_p-ERK_pp] -> [Shc_p-Grb2] + Gab1_pt + ERK_pp + [(EGF-EGFR)2_pt] | 0.2256 |  |
| 154 | [(EGF-EGFR)2_pt] -> [(EGF-EGFR)2] | 0.01 |  |
| 155 | SOS_pt -> SOS | 0.01 |  |
| 156 | Gab1_pt -> Gab1 | 0.01 |  |
| 157 | [i(EGF-EGFR)2_p-Grb2-SOS] + ERK_pp = [i(EGF-EGFR)2_p-Grb2-SOS-ERK_pp] | 0.5 | 1 |
| 158 | [i(EGF-EGFR)2_p-Grb2-SOS-ERK_pp] -> Grb2 + SOS_pt + ERK_pp + [i(EGF-EGFR)2_p] | 0.0426 |  |
| 159 | [i(EGF-EGFR)2_p-Shc_p-Grb2-SOS] + ERK_pp = [i(EGF-EGFR)2_p-Shc_p-Grb2-SOS-ERK_pp] | 0.5 | 1 |
| 160 | [i(EGF-EGFR)2_p-Shc_p-Grb2-SOS-ERK_pp] -> [Shc_p-Grb2] + SOS_pt + ERK_pp + [i(EGF-EGFR)2_pt] | 0.0426 |  |
| 161 | [i(EGF-EGFR)2_p-Grb2-Gab1] + ERK_pp = [i(EGF-EGFR)2_p-Grb2-Gab1-ERK_pp] | 0.000185 | 1 |
| 162 | [i(EGF-EGFR)2_p-Grb2-Gab1-ERK_pp] -> Grb2 + Gab1_pt + ERK_pp + [i(EGF-EGFR)2_pt] | 0.2256 |  |
| 163 | [i(EGF-EGFR)2_p-Shc_p-Grb2-Gab1] + ERK_pp = [i(EGF-EGFR)2_p-Shc_p-Grb2-Gab1-ERK_pp] | 0.000185 | 1 |
| 164 | [i(EGF-EGFR)2_p-Shc_p-Grb2-Gab1-ERK_pp] -> [Shc_p-Grb2] + Gab1_pt + ERK_pp + [i(EGF-EGFR)2_pt] | 0.2256 |  |
| 165 | [i(EGF-EGFR)2_p-Grb2-Gab1_p] + ERK_pp = [i(EGF-EGFR)2_p-Grb2-Gab1_p-ERK_pp] | 0.000185 | 1 |
| 166 | [i(EGF-EGFR)2_p-Grb2-Gab1_p-ERK_pp] -> Grb2 + Gab1_pt + ERK_pp + [i(EGF-EGFR)2_pt] | 0.2256 |  |
| 167 | [i(EGF-EGFR)2_p-Shc_p-Grb2-Gab1_p] + ERK_pp = [i(EGF-EGFR)2_p-Shc_p-Grb2-Gab1_p-ERK_pp] | 0.000185 | 1 |
| 168 | [i(EGF-EGFR)2_p-Shc_p-Grb2-Gab1_p-ERK_pp] -> [Shc_p-Grb2] + Gab1_pt + ERK_pp + [i(EGF-EGFR)2_pt] | 0.2256 |  |
| 169 | [i(EGF-EGFR)2_pt] -> [i(EGF-EGFR)2] | 0.01 |  |
| 170 | [Shc_p-Grb2] -> Shc_p + Grb2 | 3.5195 |  |
| 171 | [Shc_p-Grb2-Gab1] -> Shc_p + [Grb2-Gab1] | 3.5195 |  |
| 172 | [Shc_p-Grb2-Gab1_p] -> Shc_p + [Grb2-Gab1_p] | 3.5195 |  |
| 173 | [Shc_p-Grb2-SOS] -> Shc_p + [Grb2-SOS] | 3.5195 |  |
| **Degradation** | | | |
| 174 | [i(EGF-EGFR)2_p] -> null | 0.002 |  |
| 175 | [i(EGF-EGFR)2_p-RTKpase] -> RTKpase | 0.002 |  |
| 176 | [i(EGF-EGFR)2_p-Shc] -> Shc | 0.002 |  |
| 177 | [i(EGF-EGFR)2_p-Shc_p] -> Shc_p | 0.002 |  |
| 178 | [i(EGF-EGFR)2_p-Grb2] -> Grb2 | 0.002 |  |
| 179 | [i(EGF-EGFR)2_p-Shc_p-Grb2] -> [Shc_p-Grb2] | 0.002 |  |
| 180 | [i(EGF-EGFR)2_p-Shc_p-Grb2-SOS] -> [Shc_p-Grb2-SOS] | 0.002 |  |
| 181 | [i(EGF-EGFR)2_p-Grb2-SOS] -> [Grb2-SOS] | 0.002 |  |
| 182 | [i(EGF-EGFR)2_p-Grb2-SOS-RasGDP] -> [Grb2-SOS] + RasGDP | 0.002 |  |
| 183 | [i(EGF-EGFR)2_p-Shc_p-Grb2-SOS-RasGDP] -> [Shc_p-Grb2-SOS] + RasGDP | 0.002 |  |
| 184 | [i(EGF-EGFR)2_p-Grb2-Gab1] -> [Grb2-Gab1] | 0.002 |  |
| 185 | [i(EGF-EGFR)2_p-Shc_p-Grb2-Gab1] -> [Shc_p-Grb2-Gab1] | 0.002 |  |
| 186 | [i(EGF-EGFR)2_p-RasGAP] -> RasGAP | 0.002 |  |
| 187 | [i(EGF-EGFR)2_p-Grb2-Gab1_p-RasGAP] -> [Grb2-Gab1_p] + RasGAP | 0.002 |  |
| 188 | [i(EGF-EGFR)2_p-Shc_p-Grb2-Gab1_p-RasGAP] -> [Shc_p-Grb2-Gab1_p] + RasGAP | 0.002 |  |
| 189 | [i(EGF-EGFR)2_p-Grb2-Gab1_p-PI3K] -> [Grb2-Gab1_p] + PI3K | 0.002 |  |
| 190 | [i(EGF-EGFR)2_p-Shc_p-Grb2-Gab1_p-PI3K] -> [Shc_p-Grb2-Gab1_p] + PI3K | 0.002 |  |
| 191 | [i(EGF-EGFR)2_p-RasGAP-RasGTP] -> RasGAP + RasGTP | 0.002 |  |
| 192 | [i(EGF-EGFR)2_p-Grb2-Gab1_p-RasGAP-RasGTP] -> [Grb2-Gab1_p] + RasGAP + RasGTP | 0.002 |  |
| 193 | [i(EGF-EGFR)2_p-Shc_p-Grb2-Gab1_p-RasGAP-RasGTP] -> [Shc_p-Grb2-Gab1_p] + RasGAP + RasGTP | 0.002 |  |
| 194 | [i(EGF-EGFR)2_p-Grb2-Gab1_p-PI3K-PIP2] -> [Grb2-Gab1_p] + PI3K + PIP2 | 0.002 |  |
| 195 | [i(EGF-EGFR)2_p-Shc_p-Grb2-Gab1_p-PI3K-PIP2] -> [Shc_p-Grb2-Gab1_p] + PI3K + PIP2 | 0.002 |  |
| 196 | [i(EGF-EGFR)2_p-Grb2-SOS-ERK_pp] -> [Grb2-SOS] + ERK_pp | 0.002 |  |
| 197 | [i(EGF-EGFR)2_p-Shc_p-Grb2-SOS-ERK_pp] -> [Shc_p-Grb2-SOS] + ERK_pp | 0.002 |  |
| 198 | [i(EGF-EGFR)2_p-Grb2-Gab1-ERK_pp] -> [Grb2-Gab1] + ERK_pp | 0.002 |  |
| 199 | [i(EGF-EGFR)2_p-Shc_p-Grb2-Gab1-ERK_pp] -> [Shc_p-Grb2-Gab1] + ERK_pp | 0.002 |  |
| 200 | [i(EGF-EGFR)2_p-Grb2-Gab1_p] -> [Grb2-Gab1_p] | 0.002 |  |
| 201 | [i(EGF-EGFR)2_p-Grb2-Gab1_p-ERK_pp] -> [Grb2-Gab1_p] + ERK_pp | 0.002 |  |
| 202 | [i(EGF-EGFR)2_p-Grb2-Gab1-ERK_pp] -> [Grb2-Gab1] + ERK_pp | 0.002 |  |
| 203 | [i(EGF-EGFR)2_p-Shc_p-Grb2-Gab1_p] -> [Shc_p-Grb2-Gab1_p] | 0.002 |  |
| 204 | [i(EGF-EGFR)2_pt] -> null | 0.002 |  |
| 205 | [i(EGF-EGFR)2] -> null | 0.002 |  |
| **Production and elimination of ROS** | | | |
| 206 | PIP3 + RacGDP = [PIP3-RacGDP] | 0.002 | 0.18 |
| 207 | [PIP3-RacGDP] -> PIP3 + RacGTP | 0.4134 |  |
| 208 | RacGTP -> RacGDP | 0.262 |  |
| 209 | proNOX + RacGTP = [proNOX-RacGTP] | 0.0306705 | 0.1 |
| 210 | [proNOX-RacGTP] -> NOX | 1 |  |
| 211 | NOX -> ROS + NOX | 6000000 |  |
| 212 | NOX -> proNOX + RacGDP | 0.0141711 |  |
| 213 | null -> ROS | 200000 |  |
| 214 | ROS -> null | 991.346 |  |
| **Activation and inhibition of ASK1** | | | |
| 215 | ASK1 + ROS = [ASK1-ROS] | 0.00000001 | 0.0006 |
| 216 | [ASK1-ROS] -> ASK1_p | 0.1 |  |
| 217 | ASK1 + AKT_pp = [ASK1-AKT_pp] | 0.003 | 0.5 |
| 218 | [ASK1-AKT_pp] -> ASK1_pt + AKT_pp | 3 |  |
| 219 | ASK1_p + AKT_pp = [ASK1_p-AKT_pp] | 0.003 | 0.5 |
| 220 | [ASK1_p-AKT_pp] -> ASK1_pt + AKT_pp | 3 |  |
| 221 | ASK1_pt -> ASK1 | 0.01 |  |
| 222 | ASK1_p -> ASK1 | 0.01 |  |
| **Activation and deactivation of P38** | | | |
| 223 | MKK + ASK1_p = [MKK-ASK1_p] | 0.01 | 5 |
| 224 | [MKK-ASK1_p] -> MKK_p + ASK1_p | 3.5 |  |
| 225 | MKK_p + ASK1_p = [MKK_p-ASK1_p] | 0.01 | 5 |
| 226 | [MKK_p-ASK1_p] -> MKK_pp + ASK1_p | 2.9 |  |
| 227 | MKK_pp + MKKpase = [MKK_pp-MKKpase] | 0.0143 | 0.8 |
| 228 | MKK_p + MKKpase = [MKK_p-MKKpase] | 0.00025 | 0.5 |
| 229 | [MKK_pp-MKKpase] -> MKK_p + MKKpase | 0.058 |  |
| 230 | [MKK_p-MKKpase] -> MKK + MKKpase | 0.058 |  |
| 231 | P38 + MKK_pp = [P38-MKK_pp] | 0.001 | 1 |
| 232 | P38_p + MKK_pp = [P38_p-MKK_pp] | 0.001 | 1 |
| 233 | [P38-MKK_pp] -> P38_p + MKK_pp | 16 |  |
| 234 | [P38_p-MKK_pp] -> P38_pp + MKK_pp | 5.7 |  |
| 235 | P38_pp + P38pase = [P38_pp-P38pase] | 0.007 | 0.6 |
| 236 | [P38_pp-P38pase] -> P38_p + P38pase | 0.27 |  |
| 237 | P38_p + P38pase = [P38_p-P38pase] | 0.005 | 0.5 |
| 238 | [P38_p-P38pase] -> P38 + P38pase | 0.3 |  |
| **EGFR inhibition by specific inhibitor (inh)*** | |  |  |
| 239 | [EGF-EGFR] + inh = [EGF-EGFR-inh] | 0.00005 | 0.0000383 |
| 240 | [(EGF-EGFR)2] + inh = [(EGF-EGFR)2-inh] | 0.0001 | 0.0000383 |
| 241 | [(EGF-EGFR)2-inh] + inh = [inh-(EGF-EGFR)2-inh] | 0.00005 | 0.0000383 |
| 242 | [EGF-EGFR-inh] + [EGF-EGFR] = [(EGF-EGFR)2-inh] | 0.5005 | 0.1717 |
| 243 | [EGF-EGFR-inh] + [EGF-EGFR-inh] = [inh-(EGF-EGFR)2-inh] | 0.5005 | 0.1717 |

Definition of abbreviations used: “_p” indicates that a protein is phosphorylated; “_pp” indicates that a protein is phosphorylated at two sites; “_pt” indicates that a protein is phosphorylated to be inactive; “i” indicates that a species is internalized; “[]” indicates a complex; “=” indicates a reversible reaction; “->” indicates an irreversible reaction.

* indicates these reactions were used as virtual events to inhibit EGFR activation only.
