# Supplementary material for: Systems Biology Modeling Reveals a Possible Mechanism of the Tumor Cell Death upon Oncogene Inactivation in EGFR Addicted Cancers
Source: PLoS One. 2011 Dec 14;6(12):e28930. doi: 10.1371/journal.pone.0028930 (PMC3237568; doi:10.1371/journal.pone.0028930)
Supplement: Table S2 — Initial conditions of the computational model. (DOC) [file pone.0028930.s003.doc]

**Table S2. Initial conditions of the computational model.**

| Species name | Initial Amount (nM) | Additional description |
| --- | --- | --- |
| AKT | 444 | Serine, threonine protein kinase (also known as protein kinase B) |
| ASK1 | 200 | Apoptosis signal-regulating kinase 1 (also known as mitogen-activated protein kinase kinase kinase 5 (MAP3K5)) |
| EGF | 5 | Epidermal Growth Factor |
| EGFR | 300 | [Epidermal growth factor receptor](http://en.wikipedia.org/wiki/Epidermal_growth_factor_receptor) |
| ERK | 750 | [Extracellular signal-regulated kinases 1/2](http://en.wikipedia.org/wiki/Extracellular_signal-regulated_kinases) |
| Gab1 | 43.1 | Grb2-associated-binding protein 1 |
| Grb2 | 82.4 | Growth factor receptor binding protein 2 |
| MEK | 772 | MAP ERK kinase 1/2 (also known as MAPKK-1/2, MAP2K1/2) |
| MKK | 772 | Mitogen-activated protein kinase kinase 3/6 (MKK3/6) |
| MKKpase | 200 | Specific phosphatase of MKK3/6 |
| P38 | 750 | P38 mitogen-activated protein kinase α (p38-α), also called Mitogen-activated protein kinase 14 |
| P38pase | 35 | Specific phosphatase of P38-α |
| Pase1 | 60 | Specific phosphatase of Raf |
| Pase2 | 200 | Specific phosphatase of MEK |
| Pase3 | 35 | Specific phosphatase of ERK |
| PDK1 | 500 | Phosphoinositide-dependent protein kinase 1 |
| PI3K | 240 | Phosphatidylinositol 3-kinase |
| PIP2 | 200 | Phosphatidylinositol bisphosphate |
| PP2A | 50 | Protein phosphatase 2A (AKT phosphatase) |
| proNOX | 200 | [Nicotinamide adenine dinucleotide phosphate-oxidase (NADPH oxidase) without active Rac GTPase](http://en.wikipedia.org/wiki/NADPH) |
| PTEN | 693 | Phosphatase and tensin homolog |
| RacGDP | 95.7 | Ras-related C3 botulinum toxin substrate GTPase binding with Guanosine diphosphate (GDP) |
| Raf | 743 | RAF proto-oncogene serine/threonine-protein kinase |
| RasGAP | 93.6 | [Activator protein for Ras-like GTPase](http://en.wikipedia.org/wiki/GTPase-activator_protein_for_Ras-like_GTPase) |
| RasGDP | 95.7 | Rat sarcoma protein binding with Guanosine diphosphate (GDP) |
| RTKpase | 450 | Receptor tyrosine kinase phosphatase (EGFR phosphatase) |
| Shc | 11.5 | Src Homology 2 and Collagen Domain protein |
| SOS | 82.3 | Homolog of the Drosophila melanogaster Son of sevenless |

All species not shown in this table have zero initial conditions. Ligand (EGF) initial condition is variable.
